# Best practices for studies using digital data donation

**DOI:** 10.1007/s11135-024-01983-x

**Published:** 2024-10-08

**Authors:** Thijs C. Carrière, Laura Boeschoten, Bella Struminskaya, Heleen L. Janssen, Niek C. de Schipper, Theo Araujo

**Affiliations:** 1 Department of Methodology and Statistics, Utrecht University, Utrecht, The Netherlands; 2 Institute for Information Law, University of Amsterdam, Amsterdam, The Netherlands; 3 Department of Communication Science, University of Amsterdam, Amsterdam, The Netherlands

**Keywords:** Data donation, Digital trace data, Data quality, Local processing, Privacy preservation

## Abstract

Digital trace data form a rich, growing source of data for social sciences and humanities. Data donation offers an innovative and ethical approach to collect these digital trace data. In data donation studies, participants request a copy of the digital trace data a data controller (e.g., large digital social media or video platforms) collected about them. The European Union’s General Data Protection Regulation obliges platforms to provide such a copy. Next, the participant can choose to share (part of) this data copy with the researcher. This way, the researcher can obtain the digital trace data of interest with active consent of the participant. Setting up a data donation study involves several steps and considerations. If executed poorly, these steps might threaten a study’s quality. In this paper, we introduce a workflow for setting up a robust data donation study. This workflow is based on error sources identified in the Total Error Framework for data donation by Boeschoten et al. (2022a) as well as on experiences in earlier data donation studies by the authors. The workflow is discussed in detail and linked to challenges and considerations for each step. We aim to provide a starting point with guidelines for researchers seeking to set up and conduct a data donation study.

## Introduction

In our daily life, we leave behind large amounts of digital traces, for example, when browsing through social media, when sending an e-mail, or when making bank transactions. The term *digital trace data* refers to any data that is created because of a digital interaction (Howison et al. 2011). Digital trace data have valuable applications in social science and humanities. For example, they may complement data collected through self-reports by providing more granular measurements and by reducing social desirability and recall bias (Sen et al. 2021; Stier et al. 2020; Cesare et al. 2018; Jungherr 2018). Digital trace are collected through both *platform-centric* and *user-centric* methods (Ohme et al. 2023). In platform-centric methods, the researcher obtains digital trace data directly from the platform. Examples of platform-centric collection methods are webscraping (Breuer et al. 2020; Ohme et al. 2023) or APIs (Application Programming Interface; Biehl 2015); collaboration with digital platforms (e.g., *Social Science One*; King and Persily 2020); and buying digital trace data from data resellers (Breuer et al. 2020). Platform-centric data collection approaches present potential disadvantages, such as dependency on platforms’ willingness to share data, or high costs. Furthermore, in these methods, the people who generated the digital traces might not have consented to these data being used in research. In user-centric approaches, the researcher obtains the digital trace data through—and with consent of—the participant (e.g., through web-tracking; Ohme et al. 2023). User-centric approaches allow the researcher to obtain additional data (e.g., self-reports) from the participant. User-centric approaches present other disadvantages than platform centric approaches, such as participants’ unwillingness to share data, or - in the case of web tracking—potential reactivity of the data.

An alternative, more novel user-centric collection method for digital trace data is *data donation* (Boeschoten et al. 2022a). In data donation, participants obtain their own digital trace data and share these with the researcher. This method relies on digital platforms collecting digital trace data on their users. Due to privacy legislation, such as the *General Data Protection Regulation* (GDPR; European Union 2016) in the European Union, data controllers (i.e., any entity processing personal data, such as digital platforms) are obliged to provide a copy of all personal data (i.e., data relating to an identified or identifiable person) they collected on their users upon request by the person to whom the data pertains (Veale and Ausloos 2021; Wachter 2018). This legislation holds for digital trace data, as these are usually considered personal data. Digital platforms typically comply with the request for a data copy by providing a so-called *Data Download Package* (DDP; Boeschoten et al. 2022b). DDPs are usually.zip files that contain files with all collected data, such as usage history, content created or consumed by the individual, or inferences made by the platform (Boeschoten et al. 2021). After obtaining their DDP and providing informed consent, participants can donate (part of) the data from this DDP to the researcher. Data donation provides researchers access to digital trace data, while accounting for the disadvantages of other methods.

As a result of using DDPs, a researcher deals with a large amount of personal data. Since researchers are typically interested in specific data within the DDP, they have resorted to different approaches for (pre)-processing the data (i.e., filter and adjust the data of interest before donation) and to preserve privacy of participants (i.e., anonymizing or pseudonymizing the donated data). For example, to investigate social media use and well-being among adolescents, van Driel et al. (2022) collected complete Instagram DDPs which were de-identified prior to further analysis (Boeschoten et al. 2021). Kmetty and Németh (2022) used Facebook DDPs to study the validity of survey answers on music preferences. They de-identified the data immediately, under supervision of the participant. Other researchers use *data donation tools* in their studies. These are software that extract the data relevant to the researcher from a DDP and process these data to a predetermined output form. Using the ‘Open-Source Data Donation Framework’-software (OSD2F; Araujo et al. 2022), raw data is extracted from DDPs, but participants can delete extracted data points prior to donation, providing participants control over what is donated. Similarly, the software *Port* (Boeschoten et al. 2023) facilitates that DDPs can be processed (aggregated or de-identified) locally on the participant’s device. Only the locally processed data are sent to the researcher upon consent of the participant.

In data donation studies, researchers make choices that can affect the quality of the collected data. The Total Error Framework (TE Framework) by Boeschoten et al. (2022a) summarises sources of error that may be introduced at each stage of a data donation study. Since there are multiple error sources, multiple approaches to data donation, and the different fields of expertise needed to conduct a data donation study, the field of data donation would benefit from guidelines for data donation studies. Therefore, in this paper, we discuss best practices in data donation and introduce a workflow for researchers who are interested in conducting a data donation study. This workflow is based on the ideas of Port (Boeschoten et al. 2023), and facilitates that potential challenges and sources of error are accounted for. The potential challenges covered with our workflow were identified based on the TE framework and previously conducted data donation studies. With this workflow, our goal is to improve the quality of future data donation studies.

The outline of the paper is as follows. Section 2 provides an overview of the proposed workflow for preparing a data donation study. In Sects. 3 to 6, we discuss each step of the workflow in more detail and identify potential challenges related to that step. Section 7 links our workflow to the TE framework. Section 8 concludes the paper and provides an outlook.

## A workflow for setting up data donation studies

**Fig. 1:**
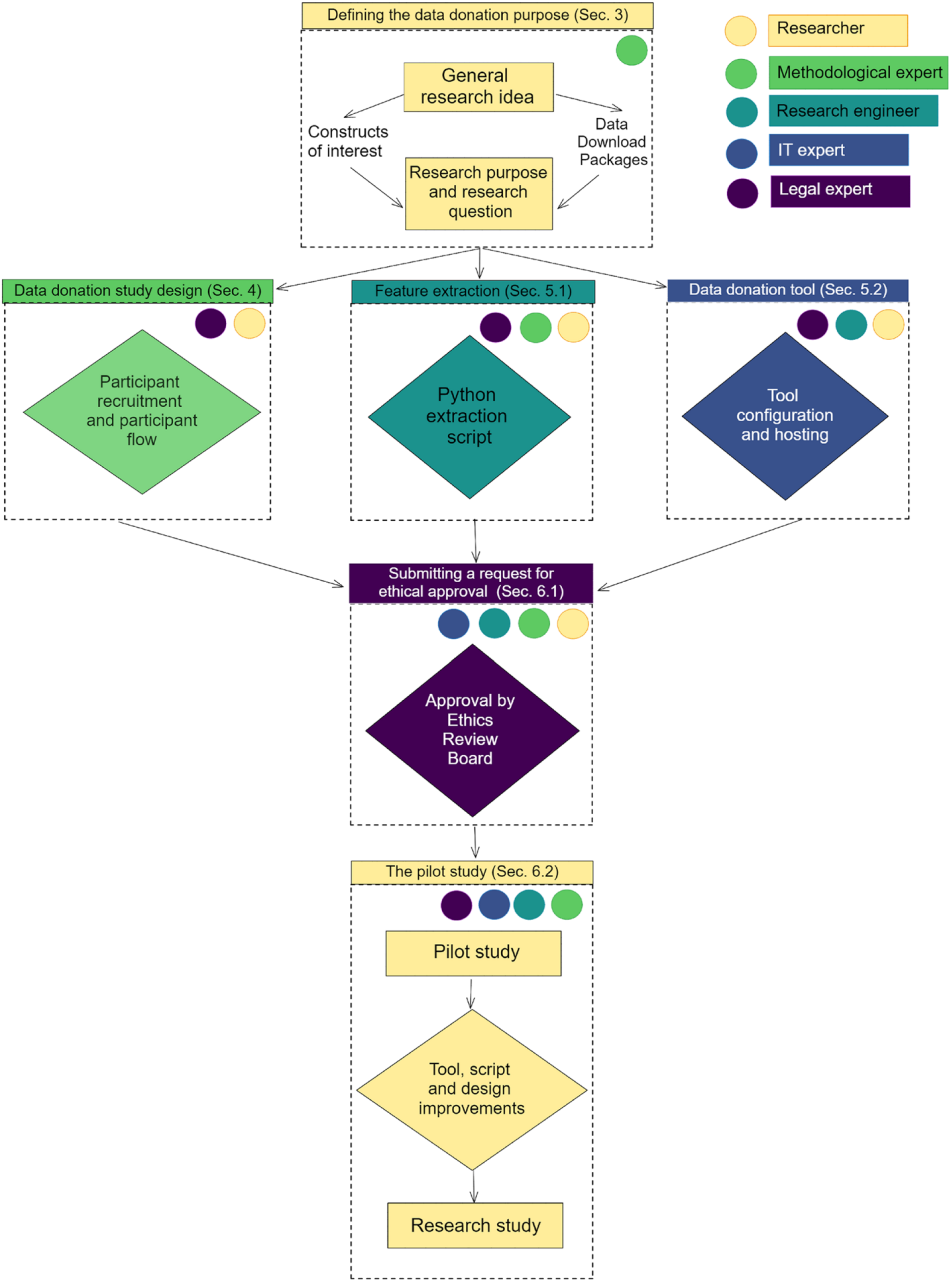
Full workflow for data donation studies. Different colors refer to the different domain experts involved. The block color indicate the primary domain expert required for this element. Colored circles indicate potential consultants. The six blocks are discussed in more detail in the subsequent paragraphs

The workflow we propose for setting up a data donation study is shown in Fig. 1. This workflow starts with a research idea or topic where the researcher considers data donation to be of added value. This research idea becomes more specific by identifying DDPs of relevant platforms and deciding which digital traces can measure the constructs of interest. The workflow continues with (simultaneously) preparing the three core elements of the data donation study: the *data donation study design*, the *feature extraction script*, and the *data donation tool*. The study is then submitted to an Ethics Review Board (ERB). After approval of the ERB, the workflow concludes with conducting a pilot study prior to fielding the main study, such that all study elements are tested and can be improved accordingly.

To successfully prepare a data donation study, unique knowledge and expertise are required from several different domains. We distinguish five domains of expertise relevant for data donation studies, represented by different roles (where a person can fulfill multiple roles depending on their expertise):

1. An *(applied) researcher* with a substantive research question to be answered through data donation is involved in all steps and carries primary responsibility for the study.
2. An *expert in the field of methodology* helps to design the study in such a way that potential bias in outcomes as a result of the study design are minimized.
3. A *research engineer* develops the script that extracts relevant features from the DDPs of interest.
4. An *IT expert* configures the server which hosts the study and data storage.
5. An *expert on data protection and legal issues* can help to ensure legal and ethical standards are met.

Figure 1 summarizes what domain expertise is required for each step of the workflow, and what other domain experts could be possible consultants.

To further illustrate the workflow and the potential challenges that can arise at each step, we introduce two illustrative research questions that can be answered through data donation. The first illustrative research question is based on a study by Struminskaya (2022)^1^:

*Did trends in travel behaviour in the Netherlands change during the Covid-19 pandemic?*       (*Illustration* 1)

Struminskaya (2022) investigated methodological aspects of willingness and consent to data donation in a Dutch non-probability panel, using data about travel as a subject matter. Data on the number of kilometres and hours travelled was extracted from Google Semantic Location History (GSLH) DDPs and processed to aggregate statistics, using Port (Boeschoten et al. 2023).

The second example research question is based on the data donation study by Corten et al. (2023):

*What are the social network structures in group chats?*       (*Illustration* 2)

Corten et al. (2023) studied behaviour of participants in WhatsApp groups, using a probability-based panel that represents the general Dutch population. They used Port to extract and aggregate both the number of WhatsApp contacts and group chats from a WhatsApp DDP, and summary statistics on a single WhatsApp group chat (e.g., number of messages sent or number of locations shared by each group chat member).

## Defining the data donation purpose

When a researcher plans to collect data, the GDPR requires the researcher to define the purpose and added value of their study (Article 29 Data Protection Working Party 2013). Crucial here is that the researcher can explain that the use of data donation within their study is a well-considered decision. For this, the researcher should think about the specific implementation of data donation in their study. We specified the following five steps that can help the researcher in making this implementation more concrete. In Table 1, we illustrate these steps for the illustrative research question of GSLH.

**1. Operationalize constructs and specify the research question:**

First, the researcher defines the construct(s) of interest. In some cases, a research question can provide further specifications regarding the context in which those constructs are measured, such as a population or timeframe. For example, for the construct ‘travel behaviour’ in illustration 1, the specifications *distinction between modes of travel*, *tracking over time*, and *commonly used in the Netherlands* can be identified (see Table 1).

**2. Select candidate DDPs based on the chosen constructs:**

In order to decide from which platform data will be donated, an overview should be created of all platforms that are related to the construct(s) under study and of its user population, including platforms that are not (well) known to the researcher. Platform usage can differ strongly over age groups, countries, and levels of education (Duggan et al. 2013). Therefore, recent sources such as national studies on platform usage (e.g. Hoekstra et al. 2022 in the Netherlands) can provide researchers directions for relevant platforms to include.

**3. Evaluate the constructs and research question for each candidate DDP:**

Once an overview of candidate platforms is created, the researcher can evaluate how well each platform matches the defined constructs and the research question. Based on this evaluation, the researcher may select a single platform to continue the study with, or may alternatively select multiple platforms when multiple DDPs are equally suited or supplementary to each other. Manzke (2024) provides an overview on factors that can be considered when selecting platforms.

**4. Re-operationalize the constructs in light of the DDPs:**

Next, the researcher determines what data from the DDPs will be used to measure the constructs (i.e., a second iteration of operationalization of the construct in light of the selected DDPs is performed). Constructs can be measured either directly or indirectly in a DDP. Direct constructs are measures taken directly from the DDP and require no processing (e.g., ‘Your likes on Instagram’). An indirect construct requires processing of the data from the DDP (e.g., *travel behavior* in Table 1). For indirect constructs, an exact alignment between the construct and the measurements obtained from the DDPs is not always possible. In case of a bad alignment, using data donation might compromise on the validity of the studied constructs.

**Fig. 2:**
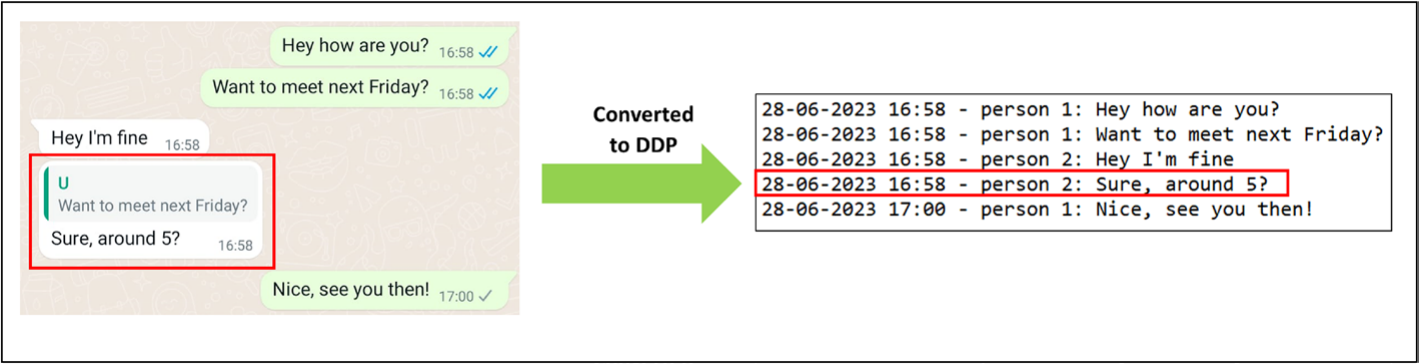
Illustration of the WhatsApp functionality to quote other user’s messages, and how data on this functionality cannot be found in the WhatsApp DDPs. Note that the data for this figure were created by the authors, no data generated by study participants is used in this example

A challenge in determining the operationalization of the construct in the DDPs, is that platforms not always provide data on all their functionalities (Carrière et al. 2023). As a result, data of interest can be missing from a DDP. The researcher should always check if the data of interest are indeed present in the DDPs. For example, WhatsApp provides no data on their functionality to quote/reply to other user’s messages (see Fig. 2). These data could have been useful to measure the construct ‘who responds most to someone’ for the WhatsApp study (illustration 2), but were unavailable.

**5. Identify and account for potential limitations of the selected DDPs:**

Common limitations of the selected DDPs to consider are conditions under which the platform does not collect data (e.g., see Table 1), platform features on which no data are present in the DDPs (see Fig. 2), and the presence of potentially incorrect measurements in the DDPs (e.g., incorrect GSLH logs as found by Struminskaya 2022). Where possible, limitations should be taken into account, as this ensures the quality of the collected data. However, some limitations may inherently be part of a platform or DDP, and it might be impossible for the researcher to account for such limitations.

Following the steps presented in this section should result in a concretized data donation idea (i.e., specified study constructs, an overview of relevant data points in the DDPs of choice, and potential limitations resulting from the choices made). Achieving this provides the researcher with a base to continue with the three core study elements. However, when following the steps, the researcher might conclude that no suitable digital trace data for the construct of interest are available. In this case, the researcher should reconsider the use of data donation in their study, as using digital traces that are unsuitable for the construct of interest would compromise on study quality and the validity of the results.

**Table 1:** Illustration on concretization of the use of data donation for illustration 1: *‘Did trends in travel behavior in the Netherlands change during Covid-19 pandemic?’*

**Step**		**Considerations for** *‘travel behavior’*			
**1.**	**Operationalize construct(s) and RQ specification**	Our construct *Travel behavior* should reflect:			
		$\bullet$ Distinction between different modes of travel			
		$\bullet$ Tracking over time			
		$\bullet$ Reflect typical behavior in the Netherlands			
**2.**	**Select candidate Data Download Packages (DDPs)**	Examples of platforms that monitor people’s movements:			
		$\bullet$ OV chip card (Dutch electronic card for public transport)			
		$\bullet$ TomTom			
		$\bullet$ Google Maps			
		$\bullet$ Google Semantic Location History (GSLH)			
		$\bullet$ Strava			
		$\bullet$ Google Fit			
**3.**	**Evaluate the candidate** $\hbox {DDPs}^1$	Platform	Modes of travel	Tracking over time	Reflect the Netherlands
		OV chip card	$\varvec{\pm }$	$\varvec{+}$	$\varvec{+}$
		TomTom	$\varvec{-}$	$\varvec{+}$	$\varvec{\pm }$
		Google Maps	$\varvec{\pm }$	$\varvec{+}$	$\varvec{+}$
		**GSLH**	$\varvec{+}$	$\varvec{+}$	$\varvec{+}$
		Strava	$\varvec{-}$	$\varvec{+}$	$\varvec{\pm }$
		Google Fit	$\varvec{-}$	$\varvec{+}$	$\varvec{\pm }$
	After step 3, it can be determined that GSLH is the preferred DDP for proceeding with the study.				
**4.**	**Operationalize constructs in DDPs**	We operationalize ’travel behavior’ by measuring every mode of travel as identified by GSLH in number of kilometers and number of hours per month.			
**5.**	**Map limitations of choices**	$\bullet$ Locations are only tracked when a Google app is present on the device and the user logged into this app with a Google account.			
		$\bullet$ Smartphone users can switch off the possibility of Google tracking their location.			
		$\bullet$ Apple devices have the tracking functionality switched off by default.			
		$\bullet$ Measurements are only provided on movements where the device is present. Participants of studies do not always bring their phone (Keusch et al. 2022), so not all their movements will be measured.			
		$\bullet$ Occasionally, Google makes mistakes in measuring activities, either by incorrect distances/times, or by linking incorrect travel modes to the movements.			

Note: $^a$In our evaluation roster, $\varvec{+}$ indicates a positive evaluation on this aspect, $\varvec{\pm }$ indicates a neutral evaluation, and $\varvec{-}$ indicates a negative evaluation

## The data donation study design

### Recruitment of participants

The data donation study design forms the first of the three study components that are worked out simultaneously in our workflow, and is worked out in collaboration of the researcher and the *expert in the field of methodology*. We identify two main elements as part of the data donation study design. The first, **recruitment of participants**, includes all actions and considerations in finding and inviting participants for the study. Choices in the recruitment of participants depend on several factors, such as the desired mode of communication, the available budget, and the platform of interest. We now discuss the elements we consider most influential for data donation.

First, data donation can be implemented in both qualitative and quantitative studies. It can serve the purpose of studying topics in more detail or help in making inferences about a population. For both purposes, literature on sampling designs, sampling strategies, and related challenges should be consulted (e.g., Lohr 2021; de Leeuw et al. 2008; Frankel 1983; Hibberts et al. 2012). Here, the researcher should be aware that participants can have or use multiple accounts on the platform of interest. Not considering a potential misalignment between the number of accounts and the number of participants can lead to incomplete data collection.

Second, reaching the target population can either be outsourced using a (non)-probability based panel, or can be done by the researchers themselves. An advantage of the former is that participants might be more familiar with the sponsor and thus more willing to participate (Keusch et al. 2019; Struminskaya et al. 2020). By considering various population characteristics, such as age distribution, (digital) literacy, and geographical distribution, the researcher can tailor the study design (e.g., by assisting participants in a lab or through online meetings, or by adjusting the available languages of study materials).

Third, the researcher may consider using incentives to increase participation. In general, monetary incentives are considered best to increase participation in survey studies (Toepoel 2012). Similar results are found for data donation studies (Silber et al. 2022; Kmetty et al. 2024), and other innovative data collection approaches (Haas et al. 2020). The desired amount differs over contexts and is still subject to research (Struminskaya 2022).

### Participant flow

**Fig. 3:**
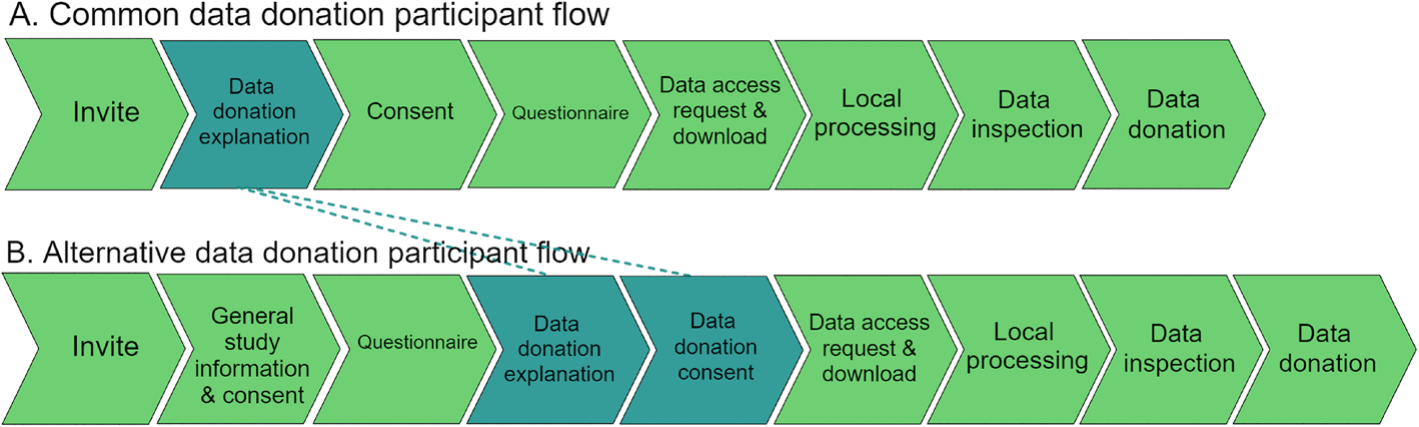
Potential variations on the order of steps in the participant flow. Dark boxes indicate the differences between the two variations

The second element of the data donation study design is the study flow from the perspective of the participant, also referred to as the **participant flow**. The participant flow of data donation studies typically consists of the following elements (see Fig. 3). First, participants receive an invitation and information letter or email, explaining the study and the data donation process. Next, a consent form is presented, after which the participant has to provide their consent in order to continue their participation. The participant flow continues with instructions on requesting and downloading the DDP at the platform of interest. Once the DDP is obtained, local extraction and processing of the data can take place. The participant can then inspect the extracted data and decide to edit or delete data points, if the researcher makes this option available. The participant flow concludes with active consent to donate the extracted data.

As data donation is a data collection approach with an active role for the participant, data donation studies are often supplemented with other data collection methods, such as questionnaires, diaries or apps, which should also be embedded in this flow. Including such additional data collection methods grants the opportunity to collect information that is hard to measure through digital trace data (e.g., opinions or psychological characteristics). Furthermore, such additional data can be used to estimate and account for potential measurement and consent error in the donated data. When including additional data collection methods, consent can be obtained separately for each method. This would lead to different variations of the participant flow, as illustrated in Fig. 3. For constructing the participant flow, we provide four recommendations:

***1. Prevent excessive burden for your participants.*** Participants might drop out of the study if the requested steps are perceived as too demanding or difficult. For example, Hase et al. (2024) found that participants indicated lack of time as a reason for not participating in Facebook data donation. Tailoring the individual elements of the participant flow to the study population can help in making the participant flow less burdensome.

***2. Clearly communicate how privacy and data protection are guaranteed.*** Rights of the participant and privacy assurance are communicated in the privacy policy and in the consent form (see Sects. 6.1.2 and 6.1.3). Since studies found that privacy concerns are negatively related to the willingness to share data (Struminskaya et al. 2020; Struminskaya 2022; Keusch et al. 2024; Pfiffner and Friemel 2023), or to participate in any data collection (Keusch et al. 2019; Sakshaug and Struminskaya 2023), privacy assurance should be stressed explicitly and repeatedly. In the WhatsApp pilot (illustration 2) and GSLH study (illustration 1), some participants perceived that their privacy was not guaranteed during the data processing and did not participate because of this. Investing in the communication of the process and the privacy during the study is therefore crucial.

**Fig. 4:**
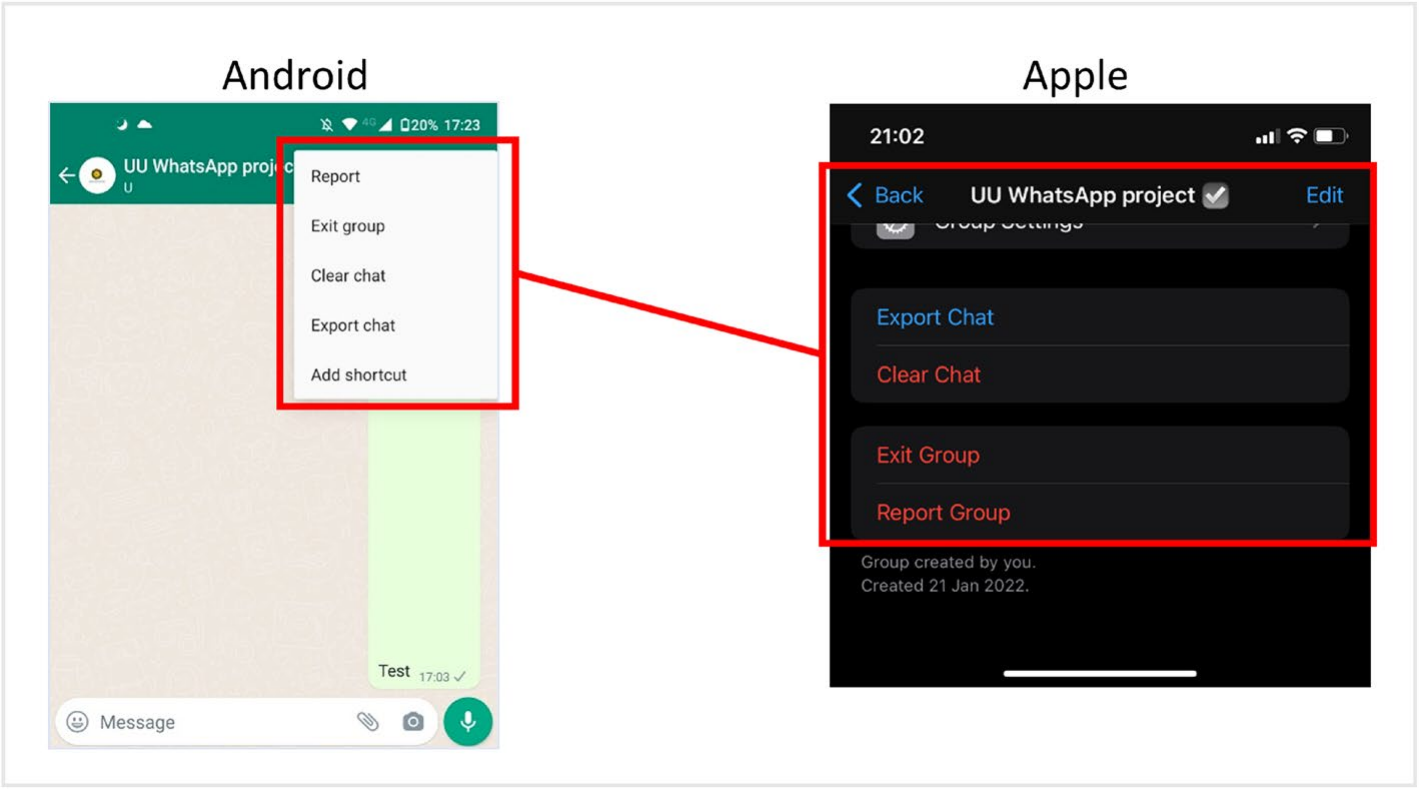
Screenshots of Apple and Android devices, illustrating that the functionality to export a chat are in different locations over different operational systems

***3. Create clear and precise instructions on how to request and download the DDP, considering the device that participants use.*** When creating the instructions for retrieving the DDPs, note that the required actions can differ over devices or operating systems (Hase et al. 2023). Figure 4 illustrates how steps in obtaining a WhatsApp chat-DDP differ between Android and Apple devices. The researcher should take potential variations in these actions into account in the instructions, and provide participants with instructions tailored to their device or operating system. In addition, platforms can differ over the devices on which DDPs can be retrieved (e.g, a Netflix DDP can only be retrieved on a computer, while the platform might be used on televisions or smartphones Hase et al. 2023). The researcher should communicate clearly to the participant what device(s) can be used during the study.

***4. Account for the time it takes for a platform to provide a DDP, and be conscious of expiration times of a DDP.*** Platforms are obliged to comply with the data access request within 30 days (European Union 2016). However, platforms differ in their time to comply, ranging from minutes to multiple days. For example, WhatsApp account information takes three days to be ready, while Google DDPs takes between an hour and three days. Communication about waiting times should be clear and correct. Well-timed reminders can help to keep participants involved in the study. Additionally, the researcher should consider expiration times of DDPs. Platforms only make DDPs available after request for a limited amount of time (Hase et al. 2023). These expiration times again differ over platforms. For example, users can download Facebook and TikTok DDPs for four days when ready, but they can download WhatsApp account information for 30 days once ready. If the researcher does not consider this expiration in the participant flow, participants might fail to obtain their DDPs and have to request them again, potentially causing these participants to quit the study.

## Data donation software

### Feature extraction

**Fig. 5:**
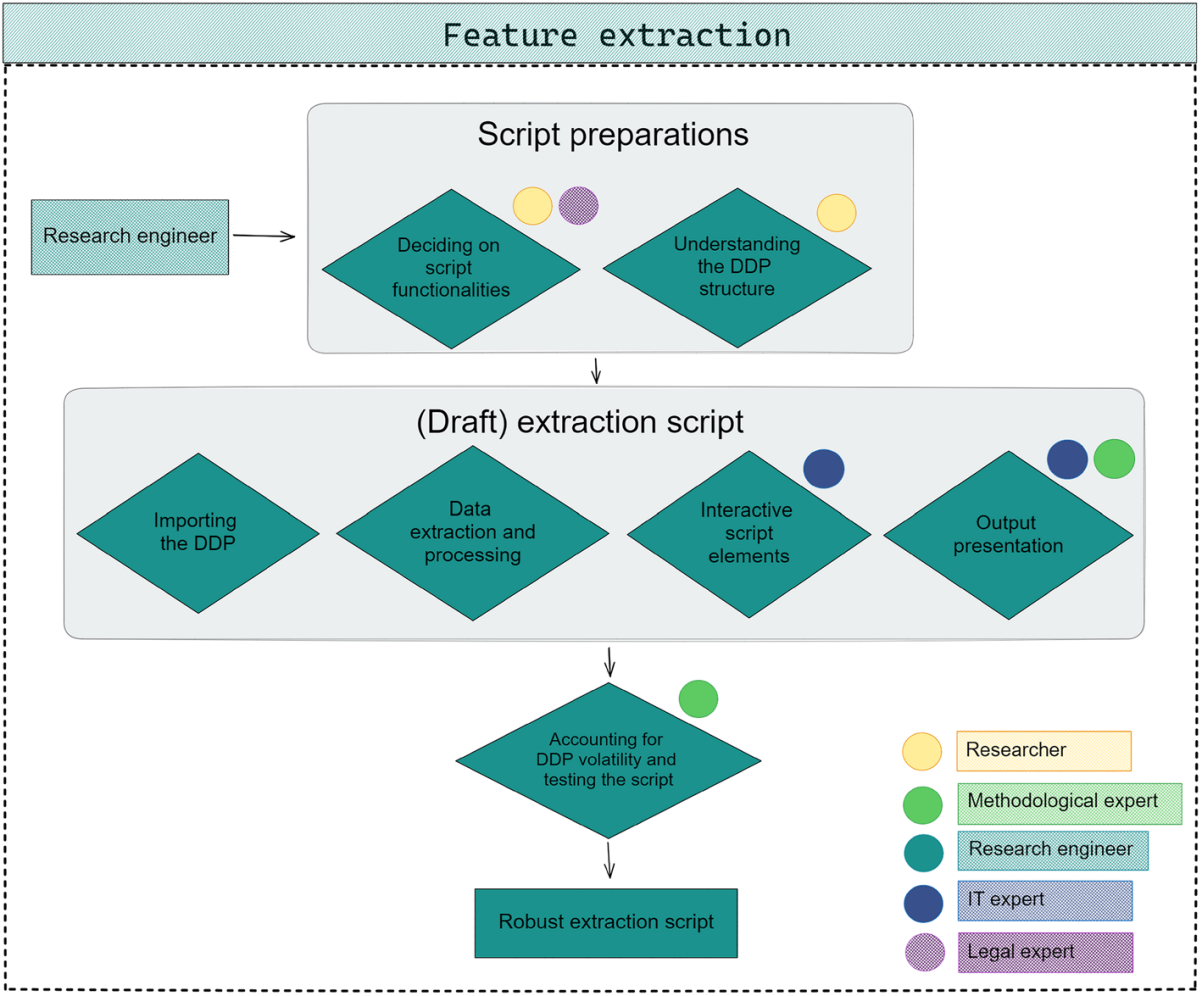
Construction of the feature extraction script. Coloured circles indicate domain experts that could be consulted for the corresponding step

For the feature extraction step, the *research engineer* builds an extraction script that locally extracts and processes the data of interest from the DDP. Although this script can be created in multiple programming languages, we illustrate the challenges encountered while building the script with a Python extraction script and with the ideas of Port (Boeschoten et al. 2023) in mind. Although for other infrastructures the technical set-up might be different, challenges and considerations are likely comparable. The process of building a robust extraction script is summarized in Fig. 5.

#### Script preparations

The preparations for building the feature extraction script entail *understanding the DDP structure* and *deciding on script functionalities*. An extraction script can only be built if it is completely clear what data needs to be extracted exactly from the DDP of interest. This should have been identified by the researcher following the steps in Sect. 3. In practice, this means that the researcher knows the exact DDP files and the locations within these files that contain data that are of interest to the study. In addition, the researcher should decide what functionalities the script needs to have. For example: In what format should the data be returned? Although a research engineer can carry out this technical work, these decisions are all the responsibility of the researcher. Once script functionalities and relevant data points are identified, the research engineer can construct a (draft) extraction script.

#### The extraction script

***Importing the DDP:***

First, the script reads in the DDP and selects the files to process. Here, consider that DDPs can appear in different file formats (e.g, a Facebook DDP can be retrieved in.json format or.html format). Furthermore, in order to make a script robust, the research engineer should think about *exception handling*. Exception handling is specifying how the script deals with unforeseen situations, such as unforeseen file types or incorrect files). Good practices for exception handling are logging meta data on occurrences of exceptions, alerting participants when unforeseen files are submitted, and ensuring exceptions do not result in errors crashing the script and aborting the donation process. The need for proper exception handling applies to all other script elements as well.

***Data extraction and processing:***

Once the DDP is read in correctly, the data of interest is extracted and potentially processed further. The level of processing differs per study, and can range from extracting raw data to using advanced data-processing algorithms. To illustrate, in the GSLH study (illustration 1), targeted data (i.e., travel logs) were processed to summary statistics (i.e., travelled kilometers per month and transportation mode) as output.

Extraction and processing may take a lot of time when complex algorithms are used or when large amount of data need to be processed. When these times are higher than anticipated by participants, participants might abort the process, assuming that a problem had occurred. To prevent this from happening, the researcher needs to clearly communicate the extraction times to the participants. This can be done either by providing information prior to extraction, or by letting the extraction script give feedback on the extraction time during the extraction process.

***Interactive script elements:***

Interaction between the participant and the script can help the researcher to extract the required data while preserving the privacy of the participants. For this, the researcher can use *interactive script elements*. Interactive script elements are steps in the processing in which input of the participant is required. For example, in the WhatsApp study (illustration 2), participants had to indicate their name from a list of all group chat participants. With this information, the script could distinguish between the data on the participant and other chat users. Without the input provided by the participant, this distinction could not have been made. To preserve the privacy of the participants, none of the names provided in the list was saved or donated.

***Output presentation:***

The final part of the extraction script summarizes the processed data and presents the output to the participant. Optionally, the researcher can decide that the participant can edit or delete part of the output presented. For example, in the study by Möller et al. (2023), participants could delete individual data points from the list of YouTube videos watched. Output can be presented in the form of tables and figures. Output presentation as graphs and charts could help participants to understand the data that they are donating (Gomez Ortega et al. 2021; Li 2021).

#### Accounting for DDP volatility and testing the script

In order to preserve the robustness of the script, the research engineer should code the script as generic as possible. One aspect which a robust extraction script should account for is the volatility in DDPs (i.e., variability in structure and content). Volatility can have the following causes (Carrière et al. 2023):

1. **The device type**. For example, in the WhatsApp study (illustration 2) we found that DDPs of Apple devices and DDPs of phones with an Android operating system differed in their notation of some data points (e.g., timestamps and sent media files).
2. **(Language) settings of the device**. In the study by Möller et al. (2023) on data donation of Twitter, YouTube, and Meta, ‘key labels’ in the.json-structures of the DDPs differed for DDPs in different languages.
3. Structures and content of DDPs can **change over time**. This happens for example when a platform adds new features or has a software update.

Unaccounted volatility can lead to the extraction script being unable to recognize the relevant data, resulting in the script not extracting the data of interest or even extracting incorrect data.

Once a first version of the script is ready, the research engineer should thoroughly test the script, using DDPs obtained through different devices and languages. In addition, good logging systems and paradata (e.g., extraction time) can provide concrete indications on script elements that need adjusting. Testing and adjusting the script form a simultaneous and iterative process that should ultimately lead to the final robust script.

### The data donation tool

For the data donation tool, the *IT expert* builds the online environment in which the data donation study can be conducted. A data donation tool consists of an app that participants can access. At the minimum, this app should allow the local processing step to take place. In practice, this means that a participant opens their DDP in the app, the extraction script is run, and after the participant presses the â€˜yes, donate’ button, the extracted data are sent to a server that the researcher can access for further analysis. To enable these steps, the researcher and IT-specialist decide where the app is hosted and where the data is stored. Hosting is possible either on premise or in a cloud. Performing a Data Protection Impact Assessment (DPIA; see Sect. 6.1.1) can guide the researcher in finding an appropriate hosting option for their study. A processing agreement should be made between the institution and the hosting party.

When a participant clicks the â€˜yes, donate’ button, the extracted data is sent to the app, which immediately sends it through to the storage account. During this process, the data are encrypted. When the data arrive at the storage location, they are no longer encrypted. Depending on the type of storage used, security measures such as multifactor authentication and role-based access control can help to ensure that only designated researchers can access this location and define the rights they have here. Again, the DPIA can help reflect on the security measures taken regarding data storage. Furthermore, a processing agreement between the institution and the party providing the data storage is required.

There are various ways to configure both the tool hosting and data storage. They can be highly tailored and making use of tools only available at the specific data collecting organizations. Alternatively, software companies can provide managed hosting solutions for researchers without technical expertise. Several initiatives on data donation tools or infrastructures are currently under development, such as the *Data Donation Module*^2^ (Pfiffner et al. 2022), the *Designerly Data Donation* platform^3^ (Gomez Ortega et al. 2021), the *ChatDashboard* software (Kohne and Montag 2023), *Dona*^4^ (Martin et al. 2023), and OSD2F (Araujo et al. 2022; also integrated in the survey tool *SocSciSurvey* by Haim et al. 2023). Although often publicly available, data donation tools are often hosted and maintained by the respective Universities, which makes it not self-evident that they can be used by researchers from other institutions. The data donation software Port (Boeschoten et al. 2023) can be used on the Next platform^5^ using service level agreements or researchers in the Netherlands can host it themselves through the national research infrastructure Surf.^6^

## Combining the core study elements

### Submitting a request for ethical approval

Based on the ideas of the three core study components, the researcher submits their study design to the Ethics Review Board (‘ERB’) of their institution. Note that this process might differ per institutions. Therefore, we recommend researchers to contact the Data Privacy Officer (‘DPO’) at their institute. However, following GDPR, most ERB submissions required a *Data Protection Impact Assessment* (DPIA), a *privacy policy*, and a *consent form*, that are guided and approved by a DPO.

#### Data protection impact assessment

A DPIA, introduced by the European Union’s GDPR (Article 29 Data Protection Working Party 2017), requires researchers to meaningfully assess and address the risks for participants created by the researcher’s data processing and provides researchers tools to manage these risks. DPIAs take the perspective of the person undergoing the processing (the participant).

DPIAs are compulsory where the processing of a participant’s personal data likely entail high risks for participants. In processing data classified as *sensitive data*, which are termed *special category data* under the GDPR, potential risks might be even higher. According to the GDPR, data revealing racial or ethnic origin, political opinions, religious or philosophical beliefs, or trade union membership, and the processing of genetic data, biometric data for the purpose of uniquely identifying a natural person, data concerning health or data concerning a natural person’s sex life or sexual orientation all concern sensitive data.

DPIAs are usually performed in distinctive, consecutive steps. First, the researcher produces documentation describing the envisaged data processing operations and the purpose(s) of the data processing. Second, the researcher motivates why they need to access and use that particular participant data, and how they limit themselves to what data is necessary to achieve the research purpose (i.e., how they adhere with data minimisation, data storage terms, keeping data accurate, and ensuring that the data is processed in confidential and secure ways). Third, the researcher assesses the potential risks to participants due to the choice of processing, whereby the researcher may ask ‘How will it affect people when the data processing works according to plan? How will people be affected when the intended data processing goes wrong? Are we allowed to process the data in this way?’, etc. Fourth, the researcher chooses appropriate mitigating technical and organisational measures (Hoofnagle et al. 2019; Demetzou 2019). Examples of technical measures are measures restricting unwanted third-party data access, authorisation techniques, using securely managed equipment, or measures securing compliance of the time period the data is stored. Examples of organisational measures are agreements with parties processing personal data on behalf of the researcher, authentication measures, or drawing up a data management plan, and getting it approved by the ERB.

It might be necessary to repeat the individual steps of the assessment, as the development of the study design progresses before any data is being processed. Example documentation for a DPIA can be found in Janssen (2023).

#### Privacy policy

A privacy policy informs participants about the collection and use of their personal data. This information should be provided to participants in a complete, concise, transparent, intelligible, easily accessible manner, and should be provided in clear and plain language. A researcher’s privacy policy should, at a minimum, inform participants about:

- the researcher’s name, and contact details of the data collecting organization and the DPO;
- the purposes of the processing for which the personal data are intended;
- the recipients or categories of recipients of the personal data (if any);
- where applicable, whether the personal data are transferred outside the EU, and a reference to the safeguards;
- the period for which the personal data will be stored;
- the existence of the right to request from the researcher access to and rectification or erasure of personal data;
- the existence of the right to withdraw consent at any time, without affecting the lawfulness of processing based on consent before its withdrawal;
- the right to lodge a complaint with a supervisory authority;
- the existence of automated decision-making, including profiling and meaningful information about the logic involved, as well as the significance and consequences of that processing for the participant (see Recitals 60 and 63); and
- where the researcher intends to further process the personal data for a purpose other than for which the data were collected, any relevant information about measures taken to secure the participant’s data (e.g., by anonymizing the personal data concerned).

Depending on the study characteristics additional information might have to be provided as well. Participants should be able to access the privacy policy at any moment during the study, but should receive all information before data collection starts. Providing the privacy policy can for example be arranged by publishing it on a website.

#### Consent & Consent form

Obtaining valid participant consent is a crucial part of legal and ethical compliance researchers must consider prior to the start of any processing of participant data. The concept of consent is traditionally linked to putting individuals whose personal data is processed in control over that processing and to build their trust and engagement in that processing.

To obtain valid participant agreement, a researchers need to obtain a participant’s *active* agreement, meaning that a participant must declare their specific and unambiguous consent before collection of their personal information or study participation can start (Neff 2008; Falagas et al. 2009). To obtain valid consent, a ‘consent form’ informing and addressing the participant’s needs and concerns must be offered, either physically or online. The information found in the consent form must fully align with the information in the privacy policy. A privacy policy is usually richer in information compared to the consent form.

For a participant’s consent to be valid, it must be *freely given, specific*, and *informed* (EDPB 2020). In general terms, any element of inappropriate pressure or influence preventing the participant from exercising their *free will*, will render the consent invalid. For consent to be *specific*, the participant must be notified about the researcher’s intentions with the data processing by specifying what kind of personal data will be processed, how that data will be used and for what specific research purpose the data will be used. Specification of the research purpose can sometimes be challenging or cannot be specified fo scientific reasons. In those cases, the GDPR allows to describe the purpose more generally. For consent to be *informed*, the researcher should provide transparently on the data processing, so that the participant can make informed choices. In data donation studies, participants give a second active consent when sharing the data after inspection, leading to a more informed consent. The researcher’s privacy policy with the minimum information elements serves this purpose.

For examples on consent in the integration of social media and survey data, see Breuer et al. (2021). Furthermore, we provide examples of consent forms in Janssen (2023).

### The pilot study

Once all study elements are ready, the researcher should conduct a pilot version of the planned study to identify potential problems that could not be found during the design phase. The researcher should account for encountered flaws or problems accordingly. If substantial changes are implemented, the researcher may consider multiple pilot iterations.

The pilot study should resemble the main study setting as much as possible in terms of participants (e.g., in general characteristics such as language and technical skill) and study flow. The researcher should furthermore aim for a variety in study situations to be included (e.g., participants with different devices or speaking different languages), so problems for different participant groups can be identified. In order to obtain more information on problems encountered by participants, we recommend that researchers survey the pilot participants about their experiences during the pilot. To obtain all desired information, the pilot study’s procedure might differ slightly from the final study’s procedure. Therefore, the researcher might have to submit a separate submission to the ERB.

The pilot study forms the first moment in the workflow where actual participant data is available to the researcher. If multiple data sources are included in the study design (e.g., both data donation and a questionnaire), the linkage of data belonging to the same participant should be arranged. Usually, linkage is ensured by including a unique person identifier per participant in each data file. The researcher should test the data linkage system on the collected pilot data. Ideally, data linkage is automated before the main data collection, as studies including large samples make manual data linkage infeasible. However, an automatic way of linking data should be created with care, as this can be error prone. Finally, the data linkage should be GDPR compliant.

## Our workflow and the Total Error framework

**Fig. 6:**
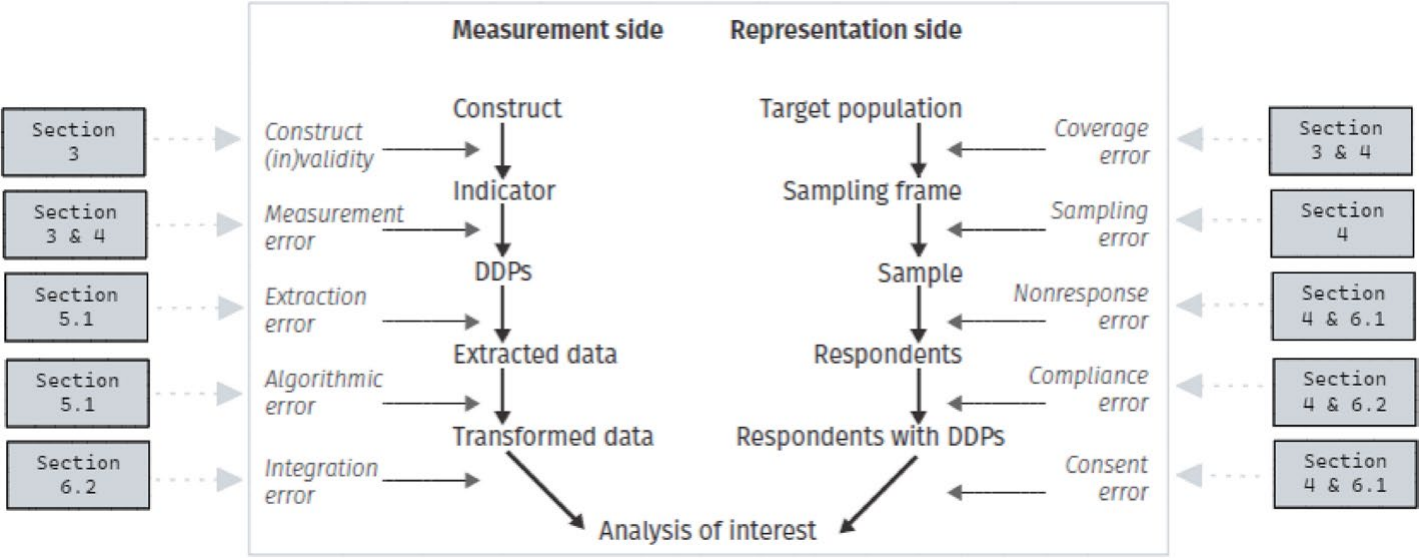
The Total Error framework for social-scientific data collection with DDPs as by Boeschoten et al. (2022a). For each error, the sections considering this error are indicated

To conceptualize potential error sources that can influence data quality, *Total Error frameworks* (TE frameworks), based on the idea of the Total Survey Error model by Groves (1989), are used (see Biemer 2010, 2016). TE frameworks have been developed for specific approaches to data collection or new data types, such as for big data (Amaya et al. 2020), digital trace data (Sen et al. 2021), and integrated data (Zhang 2012). Boeschoten et al. (2022a) designed a TE framework summarizing error sources in data donation studies. TE frameworks generally consist of a *measurement side* and a *representation side*. The measurement side comprises error sources that are introduced by the decisions on how the constructs in the study are being measured. The representation side comprises error sources related to the target population that the data is meant to represent. Figure 6 shows how our proposed workflow and the TE framework align, illustrating that the steps in our workflow together consider all errors identified by the TE framework.

### Measurement side of the TE framework

The error sources of the measurement side are considered in several of the workflow steps. Already in the phase of specifying the data donation idea (Sect. 3), decisions are made that might result in construct invalidity and measurement error. First, when the selected measurements do not align with the construct of interest, **construct invalidity** may arise. In the WhatsApp study (illustration 2), potential construct invalidity is illustrated with the construct ‘responding most to’. This construct was measured as the number of messages sent directly after another person. However, this operationalization does not consider responses to messages that were sent with other messages in between. Following the steps in Sect. 3 helps researchers with accounting for potential construct invalidity.

Second, when collected measurements for a chosen construct deviate from the true value, **measurement error** is introduced, potentially leading to biased outcome estimates. In a data donation context, measurement error is limited to error captured in the DDP before a participant obtains it, and may therefore deviate from definitions of measurement error used outside of data donation. Since the DDP data originate from online platforms, the researcher might often be unable to independently verify whether the collected measurements are free of measurement error. However, the steps proposed in Sect. 3 may help in getting an overview of the data quality provided by the online platforms and potential measurement error.

Third, when the data donation software fails to find and extract all data of interest from the DDPs, or when incorrect data are extracted, **extraction error** emerges. For example, in the WhatsApp pilot study, the script failed to extract data from DDPs with specific date-time notation, leading to incomplete data collection. In Sect. 5.1, the workflow addresses considerations to account for extraction error.

Fourth, when error arises as a result of the use of any algorithms within the extraction software, this is classified as **algorithmic error**. Algorithmic error arises in any study that makes use of algorithms, as algorithms are (almost) never perfect and make errors in classifications or inferences. For example, in the WhatsApp study (illustration 2), an algorithm could be used classify whether a message was a response on an earlier message. Not every response will be correctly classified, potentially biasing outcomes if errors are made structurally. Although the researcher cannot account for algorithmic error completely, they should study the performances of algorithms before incorporating them in the study and should not use algorithms excessively.

Last, in linking the data obtained from various sources (e.g., donated data and survey data) in the last steps of the workflow, error can be introduced, which would be classified as **integration error** (also referred to as *linkage error*; Sakshaug and Antoni 2017). We discuss considerations to account for integration error in Sect. 6.2.

### Representation side of the TE framework

The error sources from the representation side of the TE framework are linked to steps taken in the construction of the data donation study design (Sect. 4). **Coverage error** and **sampling error** both are relevant for any study where inferences to a population are made based on probability sampling. Coverage error occurs when there is a discrepancy between the target population and sampling frame (Boeschoten et al. 2022a), meaning that part of the target population is not eligible to participate in the study. In data donation studies coverage error specifically might occur when subgroups of the study population do not use the selected platform and can therefore not donate any DDP. The recommendations in Sect. 3 aim to account for discrepancies between the population of interest and study population, and subsequently for coverage error. Furthermore, sampling error emerges from observing only part of the target population in the study sample. In order to minimize the influence of both coverage error and sampling error, we recommends to seek advice from a methodological expert when constructing the study design.

The other three representation errors are linked to the participant flow. First, **non-response error** can arise when people decide not to participate in the study. Another common term describing non-response is *willingness to participate*. Depending on the exact study design, it can be more or less difficult to distinguish generic non-response from non-response caused by data donation being part of the study. Clear communication and insight in the privacy risks, and incentives can be used to achieve higher response rates and minimize non-response error. Communication about privacy considerations is also further supported by the steps in Sect. 6.1.

Second, **compliance error** arises when participants drop out anywhere in the participant flow because they cannot comply to the actions requested. For example, when unclear DDP download instruction are provided, participants with a low technologically proficiency might drop out of the study, potentially leading to biased outcomes. The steps in Sects. 4 and 6.2 account for compliance error by guiding the researcher towards detailed and correct instructions for each step of the participant flow, tailored towards the population and different operational systems and devices. Other actions to account for compliance error could be including a help desk for participants with questions and testing the participant flow, for example, through cognitive interviews (Collins 2014) to understand how participants experience the study.

Third, **consent error**^7^ emerges when participants are unwilling to donate their data. This could for example happen when the participant do not experience their privacy as guaranteed during the study. Furthermore, the visualisation of the data extracted from the DDP (Sect. 5.1.2) might aid understanding of what information is exactly sent to the researcher. If participants cannot oversee what information is exactly donated, they might tend to decline the donation. Considering these topics might help accounting for consent error.

## Conclusion and future outlook

In this paper, we introduced a workflow to guide researchers in setting up a data donation study. In the workflow, we identified five domains of expertise that are required for the different steps of the workflow. For each of the workflow steps, challenges and considerations are discussed, and ways of dealing with these are proposed. It is illustrated how the workflow steps and corresponding considerations can be linked to all error sources identified in the TE framework for data donation by Boeschoten et al. (2022a). The match between the TE framework and the proposed workflow underlines that the workflow can be used to guarantee data quality in data donation studies. To provide an overview of the recommendations and guidelines provided in this paper, Table 2 provides a summary.

The considerations and best practices provided in this paper are based on the current state of research on these topics. However, some data donation elements should be studied in more detail to achieve a better understanding of best practices. First, current data donation studies mostly revolve around social media platforms. These are often large, international platforms with an automated pipeline to handle data access requests. Smaller platforms (e.g., smaller web shops, or platforms with low numbers of users) might lack such an automated pipeline, for example due to low numbers of incoming data access requests. In studies on such platforms, it may be hard for researchers to streamline the data donation process, and more cooperation with the platform under study may be required. Second, for some of the study elements, more concrete advise could stem from further research. For example, more specific understanding on the effect of data visualisation or on the effect of monetary incentives in a data donation context would result in more specific recommendations on implementation of these topics. For both points raised, a broader use of data donation as a data collection method could help in exposing methodological challenges not encountered in current studies, and would help advance knowledge on data donation in general.

Furthermore, data donation as described in our work relies on the GDPR. More legislation around data is developing, such as the EU Data Markets Act (European Union 2022) and the recently adopted EU Data Act (European Union 2023). Such legislation might provide opportunity for more data access rights of individuals (Veale 2023). Future research could explore and discuss potential opportunities and implications of these Acts for data donation research purposes.

All taken together, data donation forms a promising and innovating approach for the collection of digital trace data. Setting up a data donation study faces challenges and pitfalls. Although some of these challenges still need to be studied more thoroughly, following the workflow and the best practices presented in this paper helps in conducting data donation studies in which the study quality is guaranteed.

**Table 2:** Overview of the recommendations and guidelines presented in this paper

Study element	Recommendations & Guidelines
Research idea	1. For relevant digital traces, consider a range of possible platforms, including potentially unfamiliar platforms to oneself
	2. Check if assumed data are present in the DDPs under study, as platforms may not provide all desired data in DDPs
	3. Be aware of specific limitations of the platforms under study (e.g., situations in which the platform does not collect data or provides incorrect data), and account for these limitations where possible
	4. When no suitable digital traces are available for the construct of interest, reconsider the use of data donation or include other data sources in the study to complement the construct
Study design	1. Create an overview on characteristics (e.g., language, technological skill) of the target population to understand how you can best help them through the study design elements
	2. Provide participants with (monetary) incentives for their participation, as data donation can be experienced as burdensome
	3. Include a questionnaire in the data donation study for information that cannot be collected through data donation (e.g., opinions, psychological traits)
	4. Make the participant flow easy to follow for all participants
	5. Make the guaranteed privacy of participants apparent, so they understand their data is safe during the study process
	6. Create exact instructions for downloading and requesting the DDP, accounting for differences in platforms and devices
	7. Account for waiting times and expiration times of DDPs, and communicate these clearly. You risk that participants are dropping out of the study if these are not accounted for
1. Feature extraction^a^	Specify the file types that the script should deal with, and specify the exception handling for unexpected file types
	2. Account for DDP volatility in the extraction script, and test the script on enough different DDPs
	3. Make the script output understandable to the participant: provide the participant with feedback on processing times and visualize the output data
Data donation tool	1. Use an established data donation tool that ensures that the data is treated as mandated by the GDPR and that you are allowed to use at your institution
Pilot study	1. Conduct a pilot study to understand how the study elements interact together, and account for problems that were not foreseen in the design phase of the study
	2. Use the pilot study to link the data of different sources (e.g., survey data and donated data) on participant level

For more concrete recommendations, see https://github.com/d3i-infra/data-donation-task/wiki/Data-donation-checklist

## Footnotes

1 Note that Struminskaya (2022) focused on the methodological aspects and we use the research question as an illustration had it been a substantive study.

2 https://datadonation.uzh.ch/en/infrastructure/.

3 https://datadonation.ide.tudelft.nl/.

4 https://dona.tf.uni-bielefeld.de/.

5 https://Eyra.co.

6 https://www.surf.nl/en.

7 Terminology as used by Boeschoten et al. (2022a). In the field of data donation, this terminology is still evolving.
